# Aggressive local treatment containing intraoperative radiation therapy (IORT) for patients with isolated local recurrences of pancreatic cancer: a retrospective analysis

**DOI:** 10.1186/1471-2407-12-295

**Published:** 2012-07-18

**Authors:** Falk Roeder, Carmen Timke, Matthias Uhl, Gregor Habl, Frank W Hensley, Markus W Buechler, Robert Krempien, Peter E Huber, Juergen Debus, Jens Werner

**Affiliations:** 1 Clinical Cooperation Unit Radiation Oncology, German Cancer Research Center (DKFZ), Im Neuenheimer Feld 280, 69120, Heidelberg, Germany; 2 Department of Radiation Oncology, University of Heidelberg, Heidelberg, Germany; 3 Department of Surgery, University of Heidelberg, Heidelberg, Germany; 4 Department of Radiation Oncology, Helios Clinic, Berlin, Germany

**Keywords:** Pancreatic cancer, Isolated local recurrence, IORT

## Abstract

**Background:**

To evaluate the use of intraoperative radiation therapy (IORT) in the multimodality treatment of patients with isolated local recurrences of pancreatic cancer.

**Methods:**

We retrospectively analyzed 36 patients with isolated local recurrences of pancreatic cancer who have been treated with a combination of surgery, IORT and EBRT. Median time from initial treatment to recurrence was 20 months. All patients were surgically explored. In 18 patients a gross total resection was achieved, whereas the other half received only debulking or no resection at all. All patients received IORT with a median dose of 15 Gy. Additional EBRT was applied to 31 patients with a median dose of 45 Gy, combined with concurrent, mainly gemcitabine-based chemotherapy.

**Results:**

Median follow-up in surviving patients was 23 months. Local progression was found in 6 patients after a median time of 17 months, resulting in estimated 1- and 2-year local control rates of 91% and 67%, respectively. Distant failure was observed in 23 patients, mainly in liver or peritoneal space. The median estimated progression-free survival was 9 months with 1- and 2-year rates of 40% and 26%, respectively. We found an encouraging estimated median overall survival of 19 months, transferring into 1- and 2-year rates of 66% and 45%. Notably 6 of 36 patients (17%) lived for more than 3 years. Severe postoperative complications were found in 3 and chemoradiation-related grade III toxicity in 6 patients. No severe IORT related toxicity was observed.

**Conclusion:**

Combination of surgery, IORT and EBRT in patients with isolated local recurrences of pancreatic cancer resulted in encouraging local control and overall survival in our cohort with acceptable toxicity. Our approach seems to be superior to palliative chemotherapy or chemoradiation alone and should be further investigated in a prospective setting specifically addressing isolated local recurrences of pancreatic cancer.

## Background

Pancreatic cancer is the fourth leading cause of cancer-related death in western countries [1]. Only about 15-20% of the patients are diagnosed in resectable stages [2]. In Europe, the standard of care for those patients consists of surgery followed by adjuvant chemotherapy [3]. But despite some improvements in overall survival by adding adjuvant chemotherapy [4], the vast majority of the patients still develops an incurable treatment failure. While in most patients the overall outcome is limited by the development of distant metastasis or combined locoregional and distant failure, about 10-35% of patients will develop an isolated local recurrence without evidence for distant spread after curative intended surgery [4,5]. While surgical resection is the only potential curative treatment approach for pancreatic cancer, and although an isolated local recurrence represents a potentially curable situation, the majority of these patients will be treated with palliative chemotherapy or chemoradiation alone. This raises the question, if a more aggressive local therapy could be beneficial at least for some of these patients. We therefore retrospectively reviewed our patients with isolated local recurrences of pancreatic cancer after primary resection, who have been treated at our institution with a more aggressive approach consisting of surgery, IORT and EBRT.

## Methods

We identified a total of 42 patients with isolated local recurrences of pancreatic cancer in the database of the University of Heidelberg, who have been treated with IORT between 2002 and 2009. Thirty-six of these patients met the premise of a curative intended treatment, which was defined as surgery without gross residual disease + IORT regardless of additional EBRT, or IORT + EBRT regardless of gross residual disease. These patients formed the basis of current analysis.

The majority of patients were male and median age at first diagnosis was 62 years. The primary treatment in all patients included surgery without macroscopic residual disease. The histological subtype was adeno-carcinoma except in three patients. The majority of patients suffered from locally advanced tumors (T3; n = 29), located in the pancreatic head (n = 29) and showed positive nodal disease (n = 21). The most frequent surgical approaches were Whipple´s procedures (n = 27). About two thirds of the patients received adjuvant chemotherapy, mainly gemcitabine-based (82%). None of the patients received chemoradiation in their primary treatment course. For detailed patient and treatment characteristics in primary situation see Table 1.

**Table 1:** Primary disease and treatment characteristics

**Primary disease and treatment characteristics**		
	**n**	**%**
age at FD		
median	62	
min	35	
max	75	
gender		
male	25	69
female	11	31
histology		
adeno-ca	33	92
other	3	8
localisation		
head	29	81
body	4	11
tail	3	8
surgery		
pp-whipple	20	56
whipple	7	19
left resection	5	14
total pancreatectomy	4	11
resection margin		
R0	21	58
R1	6	17
unknown	9	25
pT stage		
pT2	6	17
pT3	29	81
unknown	1	3
pN stage		
pN0	14	39
pN1	21	58
unknown	1	3
pTNM stage		
T2N0	3	8
T2N1	3	8
T3N0	11	31
T3N1	18	50
unknown	1	3
adj. chemotherapy		
yes	23	64
no	13	36
Gem	18	78
Gem/Cis	1	4
5-FU/LV	2	9
5-FU/FA	2	9

FD: first diagnosis, age [years], pp-whipple: pylorus-preserving whipple procedure, adj.: adjuvant, Gem: Gemcitabine, Cis: Cisplatin, 5-FU: 5-Fluororuracil, LV: Leucovorin, FA: Folinic Acid.

Local recurrence was identified by the appearance of clinical symptoms, a growing mass on repeated CT or MRI during regular follow up with a parallel increase of tumor markers or a positive PET-Scan in case of negative tumor markers. After diagnosis of a local recurrence, all patients were restaged with at least chest and abdominal CT to exclude distant metastasis. The median time interval between primary treatment and local recurrence was 20 months (range 6 to 76 months).

All patients were surgically explored and at least biopsied. In 18 patients a gross total resection was achieved, whereas the other half received only a tumor debulking or no resection at all, resulting in gross residual disease. IORT was applied to all patients either to the tumor bed or the region of gross residual disease with a median dose of 15 Gy, using a median electron energy of 8 MeV and a median cone size of 6 cm.

The technique of intraoperative radiation therapy used at the University of Heidelberg has already been described [6,7]. In brief, IORT was performed in a dedicated surgical theatre with an integrated Siemens Mevatron ME linear accelerator (Siemens, Concord, USA) capable of delivering 6-18 MeV electrons and thus covering a depth up to 6 cm. After the surgical procedure, an applicator of appropriate size was chosen to encompass the target area which was defined in correspondence with the treating surgeon. The applicator was manually positioned and attached to the table. Uninvolved radiosensitive tissues like small bowel were displaced or covered by lead shielding. The applicator was aligned with the linear accelerator using a laser guided air-docking system. The IORT dose was prescribed to 90% isodose, which covered the gross residual disease or the region of suspicious surgical margins with a safety margin of 1 cm.

**Figure 1:**
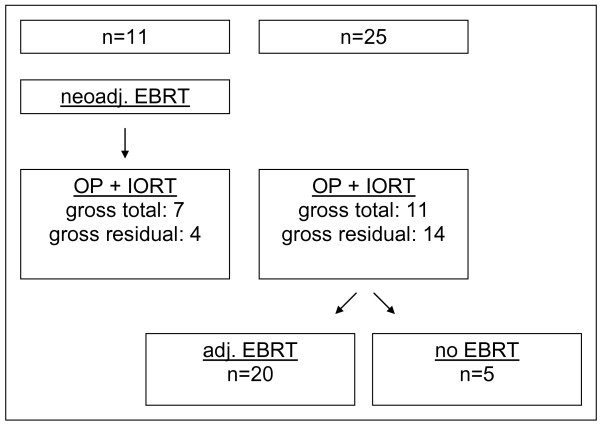
**Treatment schedule.** OP: operation, neoadj. : neoadjuvant, adj. : adjuvant, EBRT : external beam radiation therapy, IORT : intraoperative radiation therapy.

After staging, 25 patients were judged at least borderline resectable and scheduled for surgery, IORT and postoperative chemoradiation. Of these, 5 patients did not receive postoperative EBRT because of complications or patients refusal (see Figure 1a). The remaining 11 patients were initially judged as not resectable, but responded to neoadjuvant chemoradiation and were scheduled for surgical exploration and IORT (see Figure 1).

EBRT was performed by linear accelerators using 3D-conformal or intensity-modulated techniques. Patients were treated in supine position using multiple field techniques. The target volume included the gross tumor volume or the surgical tumor bed with a safety margin of 1-2 cm. No elective nodal irradiation was performed. The median EBRT dose was 45 Gy (range 39.6 to 59.4 Gy), applied in conventional fractionation (1.8-2 Gy per fraction, 5 fractions a week). EBRT was combined with concurrent chemotherapy, usually gemcitabine weekly (300 mg/sm), except in one patient. For detailed patient and treatment characteristics for recurrence see Table 2.

**Table 2:** Recurrent disease and treatment characteristics

**Recurrent disease and treatment characteristics**		
	**n**	**%**
age at rec.		
median	64	
min	37	
max	76	
time to rec.		
median	20	
min	6	
max	76	
surgery		
gross total	18	50
gross residual	6	17
Explo. lap.	12	33
IORT dose		
median	15	
min	10	
max	15	
IORT energy		
median	8	
min	6	
max	18	
IORT cone		
median	6	
min	5	
max	13	
EBRT		
neoadj RT	11	31
adj. RT	20	56
no RT	5	14
EBRT dose		
median	45	
min	39,6	
max	59,4	
EBRT technique		
3D	29	97
IMRT	2	7
conc. CHT		
yes	30	97
no	1	3
Gem	25	83
Gem/Cis	1	3
5-FU	2	7
5-FU/LV	1	3
5-FU/Cis	1	3

Rec.: recurrence, age [years], time to rec. [months], explo. Lap.: explorative laparotomy, IORT: intraoperative radiation therapy EBRT: external beam radiation therapy, neoadj.: neoadjuvant, 3D: three-dimensional CT-based conformal treatment, IMRT: intensity-modulated radiation therapy, all radiotherapy doses [Gy], conc.: concurrent, CHT: chemotherapy, Gem: Gemcitabine, Cis: Cisplatin, 5-FU: 5-Fluorouracil, LV: Leucovorin, Ox: Oxaliplatin, Erl: Erlotinib, Cap: Capecitabine, FA: Folinic Acid, Mito: Mitomycin C, Cet: Cetuximab.

Regular follow up visits took place either at our institution or at the referring center, including at least clinical examination and abdominal CT scans every three months for the first year after treatment. In case of Ca 19-9 increase or clinical evidence for local re-recurrence or distant spread, additional tests or imaging modalities were performed to confirm or exclude disease progression at the discretion of the treating physician. Acute toxicity was scored according to CTCAE V3.0, late toxicity was scored according to RTOG criteria. In case of missing follow-up examinations, data was completed by calling the patient or the treating physician. Time to event data was calculated from the first day of radiation treatment until the last follow up information or until death. Local control was defined as absence of tumor regrowth in the region of the treated recurrence on repeated CT or MRI scans. Local control (LC), Progression-free-survival and Overall Survival (OS) were calculated using the Kaplan-Meier method. In patients without further assessment of local control e.g. after development of distant spread, the date of the last information about the local status was used for calculation Differences in subgroups were tested for statistical significance by the log rank test. Differences were considered statistically significant for a p-value of ≤0.05.

The study is in compliance with the Declaration of Helsinki (Sixth Revision, 2008). Furthermore our study was approved by the Independent Ethics Committee of the Medical Faculty Heidelberg (ref. nr.: S-491/2010).

## Results

The median follow up for the entire cohort was 16 months and 23 months in surviving patients. Considering surgery, the gross total resection rate for the entire cohort was 50%. Of the 11 patients receiving neoadjuvant chemoradiation, gross total resection was achieved in 7 patients (64%). Of the 25 patients scheduled for upfront surgery, gross complete resection was achieved in 11 patients (44%), whereas 6 patients suffered from localized macroscopic residual disease (R2-situation) and in 6 patients no resection was possible at all. The difference in gross total resection rate after neoadjuvant treatment compared to upfront surgery was not statistically significant.

### Local control

**Figure 2:**
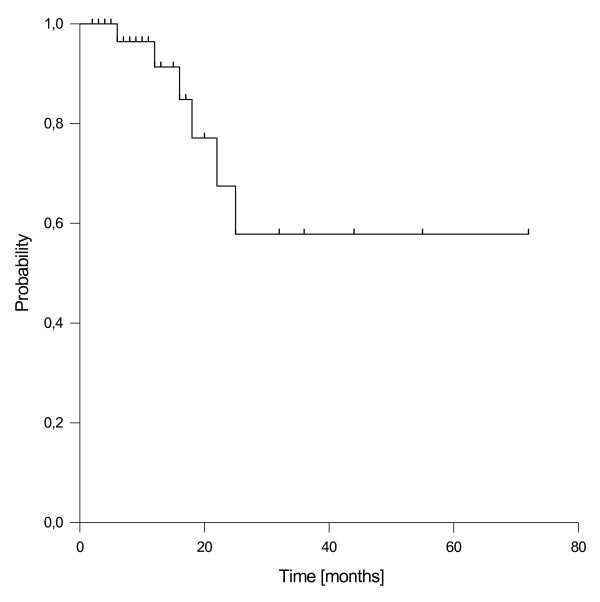
Local control.

Local recurrence/progression was observed in 6 patients (17%) after a median time of 17 months (range 7-25 months). The resulting estimated 1- and 2-year local control rates were 91% and 67%, respectively (Figure 2). Local recurrence was isolated in one patient, followed by subsequent distant failure in one patient and accompanied by synchroneous distant failure in 4 patients. All patients with a local re-recurrence have died, except one with an isolated local recurrence who remained stable with palliative chemotherapy until the last follow up visit. In univariate analysis, none of the tested prognostics factors (resection margin gross total vs. gross residual, schedule of EBRT neoadjuvant vs. adjuvant, age, gender, time to recurrence, adjuvant chemotherapy) had a significant impact on local control.

### Distant failure and progression-free survival

**Figure 3:**
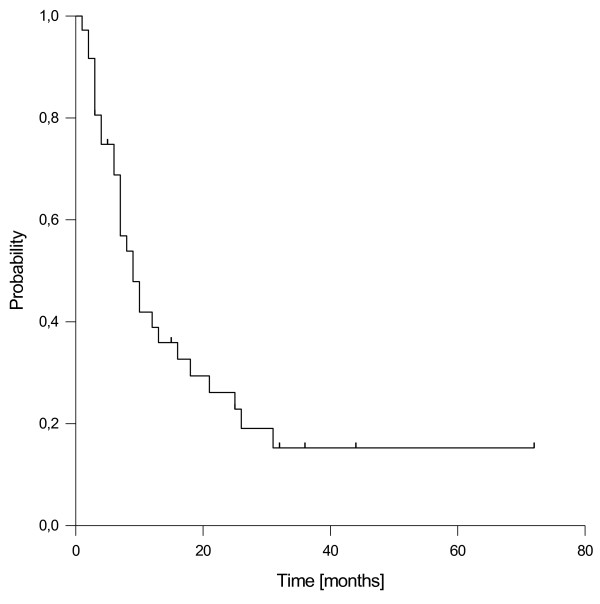
Progression-free survival.

Distant failure was observed in 23 patients after a median time of 7 months (range 1-25 months). The first site of distant failure was liver and peritoneal space in 7 patients respectively, followed by lung in 4 patients, supraclavicular nodes in one patient and combinations of these locations in 4 patients. Overall disease progression was detected in 24 patients, resulting in an estimated median progression-free survival of 9 months. The estimated 1- and 2-year rates of progression-free survival were 40% and 26%, respectively (Figure 3). None of the tested prognostic factors ((resection margin gross total vs. gross residual, schedule of EBRT neoadjuvant vs. adjuvant, age, gender, time to recurrence, adjuvant chemotherapy) showed a significant impact on progression-free survival in univariate analysis, however a trend to improved progression-free survival was observed in female patients (p = 0.058).

### Overall survival

**Figure 4:**
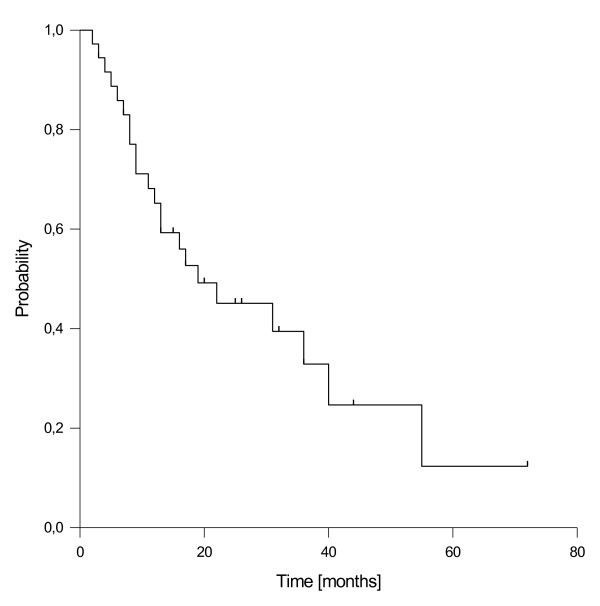
Overall survival.

Considering overall survival, a total of 22 deaths have been observed. The estimated median overall survival for the entire cohort was 19 months. The estimated 1- and 2-year rates of overall survival were 66% and 45%, respectively (Figure 4). Notably, 6 of the 36 (17%) patients lived for more than 3 years. At univariate analysis, none of the tested prognostic factors (resection margin gross total vs. gross residual, schedule of EBRT neoadjuvant vs. adjuvant, age, gender, time to recurrence, adjuvant chemotherapy) showed a significant influence on overall survival.

### Toxicity

Severe postoperative complications were observed in three patients (8%). Two patients developed intraabdominal abscess formations which resolved after re-intervention. One patient developed a perforating gastric ulcer 10 days after surgery followed by hemorrhagic peritonitis and consequently died after multiple surgical revisions, accounting for a 90-day mortality rate of 3% for the entire cohort. Minor postoperative complications, mainly wound healing disturbances and infections, were seen in 6 patients. No acute or late side effects directly related to IORT were found. Accurate data on acute toxicity during external beam radiotherapy was available in 24 of the 31 patients including complete blood cell counts for the whole treatment time in 20 of them. A total of 8 grade 3 toxicities were observed in 6 patients, mainly as gastrointestinal (nausea, vomiting, diarrhea) and hematologic side effects (see Table 3). Considering late toxicity we found severe gastrointestinal side effects in terms of diarrhea and abdominal spasms in two patients.

**Table 3:** Acute toxicity during chemoradiation

**Acute Toxicity during Chemoradiation**		
	**all grades**	**grade 3**
nausea	7	3
diarrhea	9	1
anemia	17	1
leukopenia	16	2
thrombopenia	6	1
other	3	0

## Discussion

Pancreatic cancer remains one of the diseases with the worst prognosis in oncology [2]. According to stage at presentation, patients are usually divided into three groups: resectable disease (~20%), locally advanced disease (~40%) and metastatic disease (~40%) [2]. Even in the most favourable group of patients with resectable cancer, the 5-year overall survival is limited to about 20% [4], and the vast majority of these patients still develops an incurable treatment failure, mainly distant metastasis. However, in 10-35% of the patients an isolated local recurrence without evidence for distant spread occurs [4,5]. Although an isolated local recurrence represents a potentially curable situation, little interest has been paid to specifically address this situation, no generally accepted standard treatment exists, and a special follow-up for patients after resection of pancreatic cancer is not recommended by international guidelines. Consequently these patients are treated within a variety of concepts initially developed for different stages of primary pancreatic cancer, ranging from aggressive surgical approaches as in primary resectable disease stages or chemoradiation as in locally advanced pancreatic cancer to palliative chemotherapy as in metastatic situations. However, there are some arguments for addressing this situation as a specific stage of disease. In our cohort, the median time to local recurrence from primary treatment was 20 months. In contrast to primary resectable pancreatic cancer, these patients did obviously not develop early distant metastasis, which is a common feature after curative intended surgery. For example, in the Conco-001 trial [4], which addressed the issue of adjuvant chemotherapy in resectable primary pancreatic cancer, the median disease-free survival in the experimental arm was only 13 months, mainly due to early distant failure, while local recurrence was observed only in about 20% of the patients. In contrast to metastatic disease, these patients could rationally benefit from a local treatment component as they suffer from a locoregional confined situation. Although they share some features with LAPC patients like aggressive local invasion into adjacent structures, they differ to some extent from this patient group as they have already been exposed to surgery and frequently chemotherapy. Therefore, it seems reasonable to assume (at least to some extend) a different disease biology in these patients, and to specifically address this situation as a unique stage of disease.

To our knowledge, our cohort represents one of the largest series published in peer reviewed literature that specifically addresses the treatment and outcome for isolated local recurrences of pancreatic cancer. With our treatment approach which includes radical surgery, IORT and chemoradiation, we observed encouraging rates of local control, disease-free survival and overall survival with acceptable toxicity. Notably, 6 of our 36 patients lived for more than 3 years and the estimated median survival of 19 months after the treatment of local recurrence found in our analysis compares favourable with the results published by other groups addressing LAPC (which seems to be the best available benchmark in the absence of randomized trials or larger cohort series specifically addressing isolated local recurrences). Using chemotherapy alone, modern phase III trials consistently reported median overall survival rates of only 9 to 10 months [8-10] for LAPC and about 6 to 8 months for metastatic disease [10-12]. The evaluation of chemoradiation approaches within modern phase II and III trials resulted in improved locoregional control rates but only in slightly improved median overall survival rates of 11 to 12 months [8,13-15], although some single center studies reported median overall survival rates of 17-19 months due to intensification of treatment [16-18].

Similar to primary pancreatic cancer, long term survival is hardly imaginable in patients with isolated local recurrences without the achievement of locoregional control. But even in resectable primary pancreatic cancer, local recurrences occur in up to 75% of the patients based on autopsy findings due to the always narrow and often positive margins [5]. Adjuvant chemoradiation using fractionated external beam radiation therapy (EBRT) has proven to reduce local recurrence rates compared to chemotherapy alone regardless of its conflictingly reported influence on overall survival [19]. However, the dose that can be safely applied with external beam radiotherapy is limited due to the tolerance of surrounding structures at risk, mainly small bowel. Even with the use of intensity-modulated therapy (IMRT), doses beyond 60 Gy, which are potentially needed to sterilize at least microscopic residual disease [20], can hardly be achieved without unacceptable toxicity. Especially surrounding bowel structures cannot be adaequately spared due to the narrow anatomical relations and the intra- and interfractional movements. Therefore, other radiation techniques, namely stereotactic radiosurgery (RS) and intraoperative radiation therapy (IORT) have been investigated as sole treatment or as boost techniques in combination with EBRT to achieve further dose escalation in LAPC. Notably, most of these trials also included small numbers of isolated local recurrences, but subgroup analyses were not available [21-23]. In the initial experience with RS published by the Stanford group, RS resulted in excellent short term local control rates (80-100%), but did not improve median survival (median OS 8-11 months) compared to fractionated chemoradiation and raised some concerns about late toxicity of high single doses in the pancreatic region [21,22]. However, Mahadevan et al. [23] recently reported a series of 36 patients with unresectable pancreatic cancer treated with 3 fractions of stereotactic radiotherapy up to total doses of 24-36 Gy followed by gemcitabine using an adaptive tolerance-based dose prescription approach dependent on the distance of the target to duodenal structures. After a median follow-up of 24 months, a local control rate of 78% with a median disease-free survival of 9 and a median overall survival of 14 months was found [23].

In our study, we observed comparable results (local control 83%) using intraoperative radiation therapy (IORT) as a boosting strategy in combination with maximal surgery and moderate doses of external beam radiotherapy to overcome the dose limitations mentioned above. In contrast to all external radiation therapy techniques, IORT enables the unique opportunity of displacing organs at risk from the irradiation field by simple removal during the surgical intervention. Further on, the target area is defined and treated under visual control, which minimizes the risk of a geographic miss and no additional safety margins are needed to compensate for interfraction movements. Although the applicability of the linear-quadratic model for high single doses as used in IORT has been questioned to some extend [24], it can be assumed from clinical and experimental data that a combination of 15 Gy IORT boost with 45 Gy EBRT dose as used in our study is biologically equivalent to ≥70 Gy EBRT in conventional fractionation [25]. The translation of dose escalation into improved local control found in our study has also been reported by other investigators using IORT in locally advanced unresectable pancreatic cancer: For example, Garton et al. [26] found 1- and 2-year local control rates of 86% and 68% and a median overall survival of 15 months in a series of 51 patients with unresectable pancreatic cancer treated with neoadjuvant EBRT followed by IORT. Roldan et al [27] compared patients with locally advanced pancreatic cancer scheduled either for IORT + EBRT or EBRT alone and observed a significant advantage in local control in favour of the IORT group. Mohiuddin et al. [28] found a local control rate of 69% with a median overall survival of 16 months in 49 patients with unresectable pancreatic cancer treated by IORT and postoperative chemoradiation followed by maintenance chemotherapy. Moreover, Willett et al. [29] reported a median survival of 13 months in a large series of 150 patients with unresectable pancreatic cancer treated with a combination of IORT and EBRT. These and our results compare favourable with approaches of chemoradiation or chemotherapy alone in terms of local control and strengthen the value of locally controlled disease for overall survival as supported by a recent pooled analysis on behalf of the International Society of Intraoperative Radiation Therapy (ISIORT) in Europe [30]. This described a prolonged survival in resectable pancreatic cancer patients who remained free of local recurrence for more than two years after surgery and IORT (5-years OS 28%) compared to those who failed locally (5-year OS 0%).

However, beside the encouraging local control rate found in our series, distant failure occurred in the majority of patients. In contrast to primary pancreatic cancer, the value of adjuvant chemotherapy after resection of a local recurrence or the use of additional chemotherapy after chemoradiation has not been clearly established. Therefore, only a minority of our patients received additional chemotherapy directly after completion of local treatment at the discretion of the treating medical oncologist. Given the known advantages of adjuvant chemotherapy in terms of disease-free and overall survival after gross total resection of primary pancreatic cancer [4], additional adjuvant systemic therapy might be beneficial also after local treatment of an isolated local recurrence. This should be evaluated, although a recent retrospective analysis by Baschnagel et al. [31] failed to show a benefit from additional systemic therapy after postoperative chemoradiation in primary pancreatic cancer cases. The possible value of additional systemic therapy seems to be further supported by the fact, that those studies which report encouraging results with chemoradiation for locally advanced pancreatic cancer, additional or maintenance chemotherapy was used after completion of local therapy [13,15].

Another issue in pancreatic cancer is treatment toxicity. In our study, we observed a 90-day postoperative mortality rate of 3% (1 patient). Severe postoperative complications were found in 8% of the patients. Acute gastrointestinal (GI) grade III toxicities attributable to chemoradiation were observed in 10% and grade III hematological toxicities in 20% of the evaluable patients. No severe IORT related toxicity was found. These figures compare favorable with data from the literature. Calvo et al. [32] reported 33% grade III GI-toxicity and 6% severe hematological toxicity with neoadjuvant chemoradiation + IORT. Aristu et al. [33] observed 12% severe gastrointestinal and 16% hematological toxicity during neoadjuvant chemoradiation followed by IORT in unresectable cases. IORT can be safely applied in patients with pancreatic cancer without an increase of postoperative morbidity or mortality compared to surgery alone as shown by Reni et al. [34] in a comparative series of 203 patients. Considering toxicity of external beam chemoradiation, it has to be emphasized, that severe toxicity is closely associated with the radiation technique and the irradiated volume. If modern radiation techniques are used, and the radiation fields are restricted to the gross tumor volume with small safety margins, the incidence of severe GI-toxicity appears to be much lower than in previous trials using elective nodal irradiation as well. A recent EORTC trial comparing chemotherapy with gemcitabine mono versus concurrent chemoradiation with gemcitabine in the adjuvant setting of pancreatic cancer observed no significant difference in toxicity between the treatment arms [19].

## Conclusion

In summary, to our knowledge this analysis represents the largest series published in peer-reviewed literature dealing exclusively with the treatment and outcome of isolated local recurrences of pancreatic cancer using IORT. Given the retrospective nature of our study, conclusions should be drawn with caution. However, aggressive local treatment including surgery, IORT and chemoradiation resulted in encouraging local control, median overall survival and a substantial proportion of long term survivors in our cohort with acceptable toxicity. Given the high rates of distant failure, the use of additional systemic therapy after completion of local treatment should be discussed. Nevertheless, the results with our treatment approach seem to be superior to palliative chemotherapy or chemoradiation alone and should be further tested in a prospective setting specifically addressing isolated local recurrences of pancreatic cancer.

## Competing of interest

The authors declare that they have no conflicts of interest.

## Authors contributions

FR planned the analysis, participated in data acquisition, data analysis, literature review, patient treatment, and drafted the manuscript. CT, MU, GH, FWH, RK, PH, MWB participated in data aquisition, data analysis, literature review and patient treatment. JD and JW participated in planning of the analysis, patient treatment, manuscript draft and revised the manuscript critically. All authors read and approved the final manuscript.

## Pre-publication history

The pre-publication history for this paper can be accessed here:

http://www.biomedcentral.com/1471-2407/12/295/prepub
